# Evaluation of a real-time mobile PCR device (PCR 1100) for the detection of the rabies gene in field samples

**DOI:** 10.1186/s41182-023-00501-3

**Published:** 2023-03-17

**Authors:** Catalino Demetria, Kazunori Kimitsuki, Takaaki Yahiro, Nobuo Saito, Takehiro Hashimoto, Sakirul Khan, Maria Yna Joyce Chu, Daria Manalo, Milagros Mananggit, Beatriz Quiambao, Akira Nishizono

**Affiliations:** 1 Department of Microbiology, Faculty of Medicine, Oita University, Yufu, Oita Japan; 2 Research Center for Global and Local Infectious Diseases, Yufu, Oita Japan; 3 Infection Control Center, Oita University Hospital, Yufu, Oita Japan; 4 Research Institute for Tropical Medicine, Muntinlupa City, Metro Manila Philippines; 5 Department of Agriculture Field Office III, Regional Animal Disease Diagnostic Laboratory, Tarlac, Philippines

**Keywords:** Neglected tropical diseases, Rabies, Animal rabies case, Surveillance, Philippines

## Abstract

**Background:**

The Philippines is ranked among the top countries with 200–300 annual deaths due to rabies. Most human rabies cases have been reported in remote areas, where dog surveillance is inadequate. Therefore, a strategy to effectively improve surveillance in remote areas will increase the number of detections. Detecting pathogens using portable real-time reverse transcription-polymerase chain reaction (RT-PCR) has the potential to be accepted in these areas. Thus, we aimed to develop an assay to detect the rabies virus (RABV) genome by combining the robust primer system LN34 with the PicoGene PCR1100 portable rapid instrument targeting RABV RNA (PCR1100 assay).

**Methods:**

Procedures were optimised using an LN34 primer/probe set, KAPA3G Plant PCR Kit (KAPA Biosystems), FastGene Scriptase II (NIPPON Genetics), and an artificial positive control RNA.

**Results:**

Positive control RNA showed an analytical limit of detection of 10 copies/µL without false positivity, generating results in approximately 32 min. Compared to dFAT or RT-qPCR using field samples, the sensitivity and specificity of the PCR1100 assay were 100%, and even lower copy numbers (approximately 10 copies/µL) were detected.

**Conclusions:**

This study demonstrated that the developed assay can detect rabies RNA in field samples. Because dog-mediated rabies is endemic in remote areas, the rapidity, mobility, and practicality of the PCR1100 assay as well as the high sensitivity of the LN34 system make it an ideal tool for the confirmation of rabies in these areas.

**Supplementary Information:**

The online version contains supplementary material available at 10.1186/s41182-023-00501-3.

## Background

Rabies is a neglected zoonotic disease with the highest fatality rate among infectious diseases and globally is responsible for human deaths estimated to be approximately 59,000 annually [1]. It is estimated that 99% of human deaths are due to dog-mediated rabies [2]. Inadequate surveillance data may lead to misrecognition and underestimation of the burden of rabies, which consequently decreases the priority of political will to control rabies [3–5]. The majority of rabies deaths occur in Africa (36.4%) and Asia (59.6%), and the Philippines is ranked among the top countries with 200–300 deaths annually due to rabies [6]. Most human rabies cases have been reported in remote areas, where dog surveillance is inadequate [7]. Therefore, a strategy to effectively improve surveillance in remote areas will increase the number of detections.

Direct fluorescent antibody test (dFAT), direct rapid immunohistochemistry test (DRIT), and real-time reverse transcription-polymerase chain reaction (RT-PCR) are reliable animal diagnostic methods commonly accepted by rabies diagnostic laboratories [8, 9]. The World Health Organization (WHO) and World Organization for Animal Health recommend dFAT and real-time RT-PCR as gold standard methods [1, 10], especially the LN34 primer and probe sets (the LN34 assay) initially developed by the US Centers for Disease Control and Prevention, which have addressed the need for a single TaqMan-based assay capable of detecting highly variable rabies virus (RABV) and other lyssaviruses [11]. This has been subsequently validated and has shown robustness and reliability, as this assay can also test samples that cannot be tested using dFAT, such as archived and degraded tissues [12]. However, commercially available real-time RT-PCR devices are expensive because they are equipped with a fluorometer and a thermal cycler, and they are time-consuming because they require amplification for more than one hour. Furthermore, these PCR devices are unsuitable for field operations because they are bulky and require a laboratory, preventing their application for disease diagnosis in remote areas.

Lateral flow devices (LFDs) are rapid, cost-effective, and relatively easy-to-use point-of-care (POC) tests and have the potential to decentralise laboratories by avoiding the transport of samples. Several studies have validated this tool for rabies diagnosis [13–22]. However, as it has not yet been approved as a preliminary test, LFDs must be supplemented by other diagnostic platforms to improve case detection.

In the past few years, portable PCR devices suitable for use in the field or as POC tests have been developed [23–29]. These mobile PCR devices are rapid, energy-efficient, and as sensitive as conventional real-time PCR devices [30]. The thermal cycling process in commercial real-time PCR equipment uses Peltier heaters, which require approximately 2–3 min for each cycle [30]. In contrast, microfluidic PCR technology effectively reduces the thermal cycling time because the ramping (heating and cooling) is independent of the heat capacity of the reaction vessel or the temperature-controlled metal block. PicoGene PCR1100 (Nippon Sheet Glass, Tokyo, Japan) uses a microfluidic approach, in which the solution reciprocates between heaters at different temperatures [31]. Shirato et al. used this device to develop ultra-fast and sensitive diagnostic tests for Middle East respiratory syndrome-related coronavirus, orthopneumovirus, and severe acute respiratory coronavirus 2 [31–33].

Thus, the technique of detecting pathogens using portable real-time RT-PCR has the potential to be accepted in rural facilities because its implementation is easy and practical. Therefore, we aimed to develop an assay to detect the RABV gene by combining the robust primer system LN34 with the PicoGene PCR1100 portable rapid instrument targeting RABV RNA. This device responds to concerns common to neglected tropical diseases, such as rabies, as it allows for screening and bedside diagnosis of infectious diseases. It also complements POC tools such as the LFD. This will be helpful for decision-making and as a surveillance tool in cases where a preliminary test is unavailable or inaccessible.

## Methods

### Ethical statement

Specimens collected from the Regional Animal Disease Diagnostic Laboratory (RADDL) III for national surveillance were used in this study. We kept personal information confidential and obtained verbal informed consent from the participants. In addition, as the samples were collected from carcasses submitted by local residents and organisations, approval from the Animal Ethics Committee regarding animals was not required. For biosafety clearance, the research plan was approved by the Research Institute of Tropical Medicine biosafety clearance (No. 190116). For animal experiments, mouse samples used in previous experiments were approved by the Animal Ethics Committee of Oita University (No. 191002).

### RNA and samples

An artificial positive control RNA for LN34 was developed for the assay based on previous studies [12, 34]. This RNA was used as a positive control and a standard curve was generated. RNA extracts from 10 archived mice brains, salivary glands, and muzzle skin infected with street RABV strain 1088 [35] were used as experimental samples. Mice were infected with strain 1088 from the peripheral tissue (right hind limb) and euthanised 11 days after inoculation following neurological symptoms. The samples submitted to the RADDL of region III (Central Luzon) in the Philippines for confirmatory testing were used as field samples. Samples with insufficient volume were excluded from the study. All field samples were obtained from domestic dogs or cats and tested using dFAT. The parts of the brain tested for dFAT included Ammon’s horn, medulla, and cerebrum using fluorescein isothiocyanate-conjugated anti-rabies monoclonal antibody (Fujirebio, Malvern, PA, USA) according to the standard operating procedure [9, 36]. Out of 49 samples, the number of positive and negative samples were 24 and 25, respectively (Additional file 1). The samples were stored at − 80 °C until use.

### RNA extraction

The RNeasy Mini Kit (Qiagen, Valencia, CA) was used for RNA extraction from experimental samples. Each tissue was homogenised with bead beats, and the total RNA was isolated and purified according to the manufacturer's instructions. Fifty µL of purified RNA was stored at − 80 °C until use. For field samples, total RNA was extracted from frozen medulla samples using a High Pure RNA Tissue Kit (Roche Molecular Biochemicals, Manheim, Germany). Briefly, tissue weighing approximately 30 mg was homogenised using a Precellys tissue homogeniser (Bertin Instruments, Frankfurt, Germany) and RNA was extracted following the manufacturer’s instructions. The eluted RNA (50 µL) was stored at − 80 °C until use.

### Real-time RT-PCR using conventional real-time RT-PCR equipment

The LN34 assay was performed for conventional real-time RT-PCR using AgPath-ID One-step RT-PCR Reagents (Applied Biosystems, Waltham, MA, USA) according to available publications [8, 11, 37]. Based on the previous report, a total of 25 µL reaction volume was used for this assay [37]. In details, 6.5 μL of ddH_2_O, 12.5 μL of 2 × RT buffer, 1 μL of 25 × RT PCR Enzyme Mix, 1 μL of either LN34 or β-actin primer set (10 μM), 1 μL of either LN34 or β-actin probe (5 μM), and 2 μL of RNA template. Samples were tested using a CFX connect Real-Time System or CFX96 Touch Real-Time PCR System (Bio-Rad Laboratories, Inc., Hercules, CA, USA) under the following conditions: reverse transcription at 50 °C for 30 min and denaturation at 95 °C for 10 min followed by denaturation 95 °C for 15 s and annealing/extension at 56 °C for 20 s for 45 cycles.

### Real-time RT-PCR using PicoGene PCR1100 (PCR1100 assay)

**Fig. 1:**
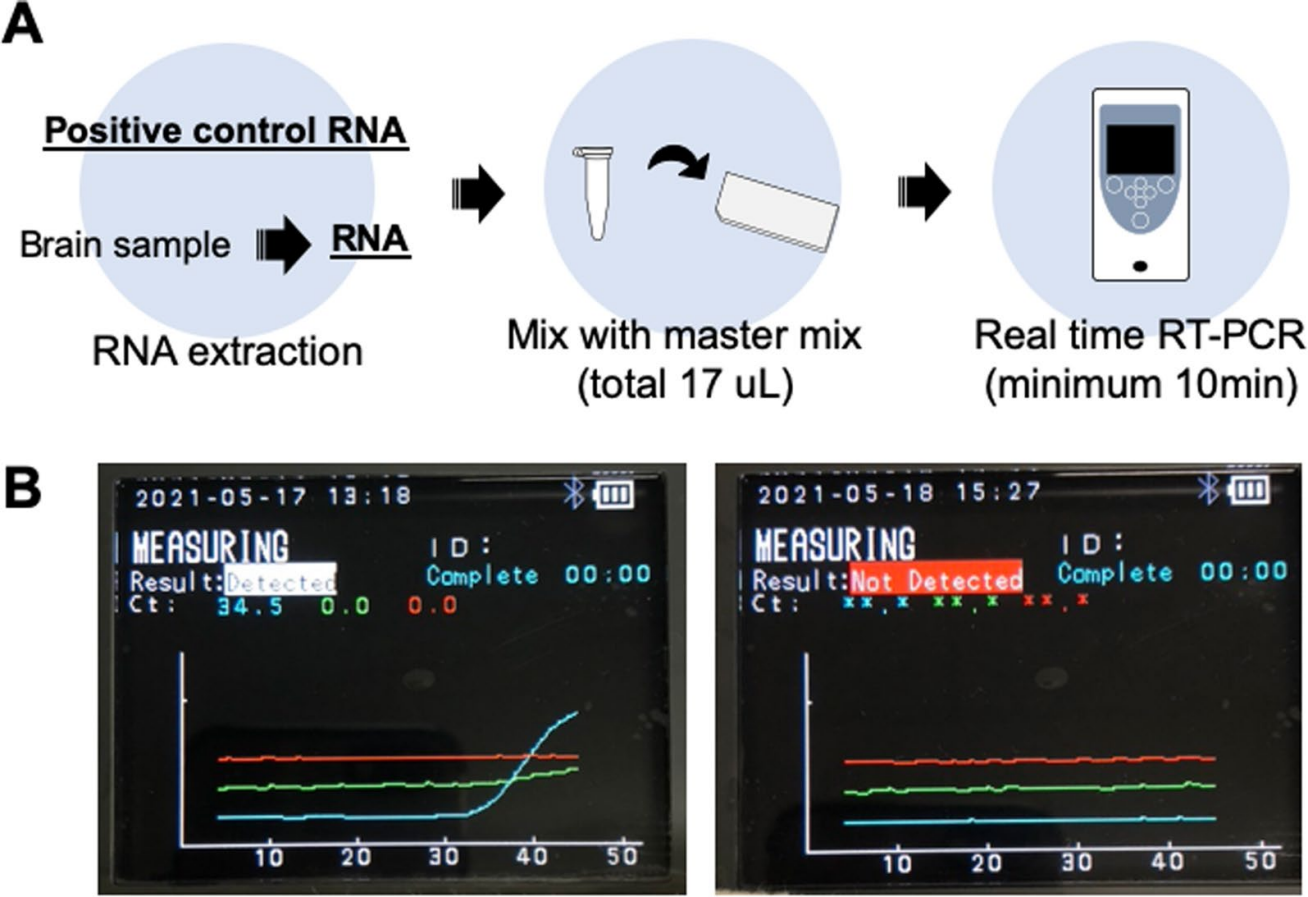
Overview of the PCR1100 assay. **A** Schematic illustration of the workflow of the real-time RT-PCR procedure used in this study. **B** Once the PCR reaction is complete, the amplification curve and results are displayed on the monitor. The left panel shows a positive result, whereas the right panel shows a negative result. Blue, green, and red lines indicate FAM, VIC, and Cy5 fluorescence, respectively

A schematic representation of PCR1100 assay is shown in Fig. 1. The One-step real-time RT-PCR assay was conducted using a KAPA3G Plant PCR Kit (KAPA Biosystems, Wilmington, MA, USA) and FastGene Scriptase II (NIPPON Genetics, Tokyo, Japan). It was optimised using a total volume of 17 µL based on previous studies [31–33]. Briefly, 0.27 µL RNase-free water, 8.5 µL, 2X KAPA3G Plant Buffer (0.4 mM dNTPs and 1.5 mM MgCl_2_), 1.15 µl of MgCl_2_ (25 mM), 3.08 µL of Primer/Probe mix, 1 µL), 1 µL DNA Polymerase, and 1 µL FastGene™ Scriptase II were mixed for a single tube (Table 1). We used LN34 and β-actin primers and probes, respectively, in a manner similar to conventional real-time RT-PCR. LN34 primer (1.2 µL each) and probe (0.68 µL) were mixed for the Singleplex assay. For the multiplex assay, primers and probes for LN34 and β-actin were mixed to a volume of 0.308 µL per reaction (Table 1). A sample template was added to the tubes, and 17 µL of this mixture was pipetted into the chip. The chip was inserted into the device to begin the test. The following conditions were used for the PCR1100 device: 42 °C for 180 s, 95 °C for 15 s, 95 °C for 5 s, and 56 °C for 30 s, which was programmed to run for 50 cycles.

**Table 1:** Components of mixture for rabies PCR1100 assay

For Singleplex assay			
Component	Concentration	Volume	
RNase-free water		0.27	µL
2 × KAPA3G Plant Buffer		8.5	µL
LN34 Forward	709 nM	1.2	µL
LN34 Reverse	709 nM	1.2	µL
LN34 Probe	200 nM	0.68	µL
MgCl_2_ (KAPA3GPlant)		1.15	µL
DNA Polymerase (KAPA 3GPlant)		1	µL
FastGene™ Scriptase II		1	µL
RNA template		2	µL
		17	µL

For Multiplex assay			
Component	Concentration	Volume	
RNase-free water		0.27	µL
2 × KAPA3G Plant Buffer		8.5	µL
LN34 Forward	497 nM	0.84	µL
LN34 Reverse	497 nM	0.84	µL
LN34 Probe	140 nM	0.476	µL
β-actin Forward	212 nM	0.36	µL
β-actin Reverse	212 nM	0.36	µL
β-actin Probe	60 nM	0.204	µL
MgCl_2_ (KAPA3GPlant)		1.15	µL
DNA Polymerase (KAPA 3GPlant)		1	µL
FastGene™ Scriptase II		1	µL
RNA template		2	µL
		17	µL

### Statistical analysis

Spearman’s correlation tests between the devices were performed using GraphPad Prism 8 (GraphPad Software, CA, USA). Total of 24 positive samples were analysed.

## Results

### Optimisation of real-time RT-PCR using PCR1100

**Fig. 2:**
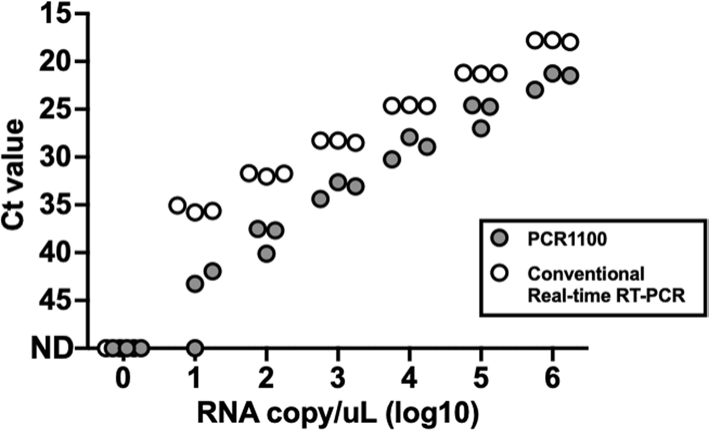
Limit of detection (LOD) analysis of the PCR1100 assay and conventional real-time RT-PCR. RABV-positive control RNA with a known copy number was tested in triplicate to determine the LOD for both the PCR1100 assay and conventional real-time RT-PCR (CFX connected Real-Time PCR). Protocols for LN34 primer/probe sets were used for the PCR1100 assay and conventional real-time RT-PCR (Singleplex assay)

We first evaluated the sensitivity of the PCR1100 assay using an LN34 artificial control. Both PCR1100 and conventional real-time PCR had a detection limit of 10 copies/µL, and the Ct value of PCR1100 was higher than that of conventional one (Fig. 2). Following the optimisation of the Singleplex assay, the assay was developed in a multiplex format. We tested brain tissue, muzzle skin, and salivary gland samples from six experimentally infected mice using multiplex assays with LN34 and β-Actin. The assay detected the target rabies RNA nucleocapsid in the brains of all six infected mice and successfully amplified the β-actin gene in all positive and negative samples. The RABV gene was detected in all of the six samples of the muzzle skin and only three of the six samples of the salivary glands (Additional file 2).

### Evaluation using field samples

**Fig. 3:**
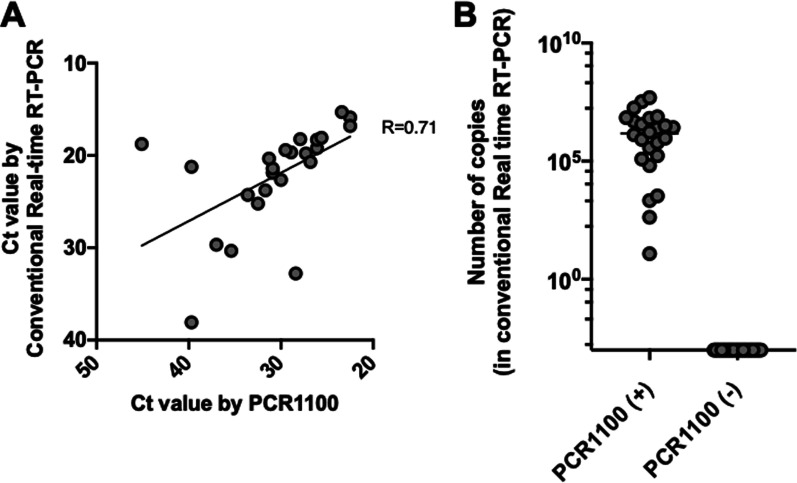
Comparison of the PCR1100 assay with conventional Real-time RT-PCR using field samples. **A** The Ct value of the PCR1100 assay was compared to that obtained using conventional real-time RT-PCR (CFX96 Touch) across a panel of 24 RABV-positive field samples. **B** The results (RABV-positive and -negative) determined by PCR1100 (x-axis) and the viral RNA copy number in each specimen determined by conventional real-time RT-PCR (y-axis) are plotted

To further determine the utility of the PCR1100 assay, canine brain samples from RADDL III were tested. The PCR1100 assay had 100% sensitivity and specificity compared to the gold standard test, dFAT, and there was 100% similarity with the results of conventional real-time PCR systems (Table 2). Among the positive samples, the Ct values between the PCR1100 assay and CFX96 Touch were correlated (*R* = 0.71) (Fig. 3A). Regarding copy number, the PCR1100 assay detected even low copy numbers (copy number = 12.2) (Fig. 3B).

**Table 2:** Sensitivity and specificity in the field samples

		dFAT		Conventional real-time RT-PCR (CFX96 Touch)		
		+	−	+	−	Total
PCR1100	+	24	0	24	0	24
	−	0	25	0	25	25
	Total	24	25	24	25	49

*dFAT* direct fluorescent antibody test

## Discussion

Rabies has been targeted for global eradication by 2030, and diagnostic tools play an important role in quantifying the disease burden needed to meet the target and monitor its sustainability. Although data on animal rabies cases are essential for designing effective countermeasures, many developing countries lack surveillance information. Particularly in rural areas, these data are severely lacking, and it is assumed that the distance to the diagnostic laboratory is the reason for this [7]. Therefore, it is necessary to develop an easy and rapid diagnostic method for remote areas. In this context, several studies have evaluated LFDs and demonstrated their functionality. However, in some situations, LFD yielded false-negative results. Furthermore, there are no national-level criteria for using LFD to diagnose rabies, and some commercially available LFDs have markedly low sensitivity and specificity [18, 36]. The mobile PCR device, PCR1100, is less expensive than conventional PCR instruments (about one-fifth the cost of a conventional real-time PCR device) and can potentially be used in the field as long as a battery is available. Moreover, in our experience, the total working time (RNA isolation, reagent preparation, and performing PCR) for the conventional real-time PCR and the PCR1100 assay is approximately 180 and 90 min, respectively. Thus, the PCR1100 assay can be diagnosed more than one and a half hour earlier than the ordinary real-time RT-PCR.

In this study, the sensitivity and specificity of the PCR1100 assay were both 100%, which is equivalent to those of dFAT and conventional real-time RT-PCR. Therefore, we suggest that this assay has potential as a supplementary diagnostic method for simple diagnostic techniques, such as LFD. The advantage of DRIT and LFD is that any front-line laboratory can efficiently perform simple diagnostics. However, DRIT has a limited availability of monoclonal antibodies and cannot be used for decomposed samples, and LFD rarely shows low sensitivity depending on the animal species and commercial product [14, 18, 36, 38, 39]. Nucleic acid detection technologies, such as RT-PCR, can be used with decomposed samples and are more sensitive and specific than dFAT and DRIT [39, 40]. In this study, the PCR1100 assay showed comparable results to conventional real-time RT-PCR for the positive control RNA, experimental samples, and field samples. In addition, the results of the PCR1100 assay for the field samples were identical to those of dFAT. This implies that we can detect positive samples without any inaccuracy, and it can support other POC tests used for screening in areas where primary tests are unavailable for confirmatory testing or laboratories are not accessible.

Park et al. showed that the RABV antigen is particularly abundant in the follicle sinus complex (FSC) in rabies-infected animals and proposed that the sinus hair in muzzle skin can be utilised as an alternative diagnostic tissue [41–43]. Based on previous reports, we were able to detect the RABV gene in laboratory samples of muzzle skin as well as the brain. Although the differences in animal species, virus strains, and sample processing methods should be considered, our study provides evidence that the RABV gene is detectable in FSCs. In contrast, the RABV gene was detected in three out of six salivary gland samples, suggesting that the detection rate was not very high. Moreover, we were unable to detect the RABV gene in 100% of samples (1 out of 3) if the virus loads are 10^1^copy/uL. One of the possible reasons for such discrepancy is due to different reaction volumes and reagent concentrations. The WHO manual for rabies diagnosis recommends to use Ag-Path ID One-Step RT-PCR Kit (Life Technologies) for the LN34 assay [8], however, we could not detect the RABV gene using PCR1100 assay with this kit (data not shown). Therefore, we used the KAPA3G Plant PCR Kit (KAPA Biosystems) and FastGene Scriptase II (NIPPON Genetics) as reported previously [31–33]. In addition to the factors related to the uses of different kits, we cannot exclude the possibility of the features of microfluidic system which may have influenced the performance. Future studies with more control samples will be used to clarify the exact low copy number detection rate.

The LN34 system included in our study is a pan-lyssavirus assay capable of detecting all known lyssaviruses, including highly divergent bat-related strains. Therefore, our assay may be useful for the global surveillance of RABV in domestic and wild animals, including bats [12].

This study had some limitations. First, RNA extraction from samples is required in laboratories. PCR should be conducted in a clean environment to avoid contamination. Therefore, we need to consider rapid RNA extraction methods using simple RNA extraction kits, such as the M1 Sample Prep Cartridge Kit for RNA (Biomeme, Philadelphia, PA, USA) [33] or direct RT-PCR [44–47]. Second, this method is unsuitable for processing multiple samples simultaneously because the PCR1100 assay can only test one sample in a single run. However, in the Philippines, where the current research is being conducted, only a few specimens are tested daily; therefore, handling them seems feasible. Moreover, we believe that the system can be used more efficiently by combining it with simple diagnostic methods, such as LDF. It can also be used to confirm false-negative dFAT and LFD results from samples submitted under poor conditions. Third, this study did not conduct a multi-site evaluation. The field samples used in this study were frozen in a rabies-endemic area in the Philippines. This process was performed by skilled technicians at a national reference laboratory. We believe that it is necessary to investigate the conditions under which this can be performed for more practical use.

## Conclusions

The PCR1100 assay used in this study is a useful tool to detect the RABV gene in a shorter time that is consistent with the gold standard. Although the PCR1100 device has some limitations, such as test samples and positive controls cannot be run simultaneously or extremely low viral load may cause false-negative results, it can be ideal for the resource-limited remote setting because a portable battery is available. Moreover, in terms of installation cost, the PCR 1100 device is thought to be an economical alternative to conventional real-time PCR. Therefore, currently reported simple diagnostic methods such as LFD combined with portable PCR devices can contribute to accurate diagnosis and surveillance in areas far from the diagnostic laboratory.

## Supplementary Information

**Additional file 1: ** Characteristics of 49 animals.

**Additional file 2: **  Diagnostic results of experimentally infected mouse samples for different tissue.

## Data Availability

The datasets used and/or analysed during the current study are available from the corresponding author upon reasonable request.

## Abbreviations

dFAT: Direct fluorescent antibody test
FITC: Fluorescein isothiocyanate
LFD: Lateral flow device
RADDL: Regional Animal Disease Diagnostic Laboratory
POC: Point-of-care
RT-PCR: Reverse transcription-polymerase chain reaction
