# Supplementary material for: Evaluation of a real-time mobile PCR device (PCR 1100) for the detection of the rabies gene in field samples
Source: Trop Med Health. 2023 Mar 17;51:17. doi: 10.1186/s41182-023-00501-3 (PMC10020757; doi:10.1186/s41182-023-00501-3)
Supplement: Supplementary file 1 — Additional file 1: Characteristics of 49 animals. [file 41182_2023_501_MOESM1_ESM.docx]

Additional file 1: Characteristics of 49 animals

|  |  |  |  | dFAT | |
| --- | --- | --- | --- | --- | --- |
| Category |  | Total | (%) | Positive | Negative |
| Species | Dog | 43 | (87.8%) | 23 | 20 |
|  | Cat | 6 | (12.2%) | 1 | 5 |
| Age | 0-1 y.o. | 32 | (65.3%) | 15 | 17 |
|  | 1-2 y.o. | 3 | (6.1%) | 2 | 1 |
|  | ≧3 y.o. | 8 | (16.3%) | 3 | 5 |
|  | Unknown | 6 | (12.2%) | 4 | 2 |
| Sex | Male | 25 | (51.0%) | 8 | 17 |
|  | Female | 14 | (28.6%) | 11 | 3 |
|  | Unknown | 10 | (20.4%) | 5 | 5 |
| Specimen | Whole carcass | 7 | (14.3%) | 2 | 5 |
|  | Head | 42 | (85.7%) | 22 | 20 |
| Cause of Death | Euthanasia | 7 | (14.3%) | 5 | 2 |
|  | Illness | 34 | (69.4%) | 13 | 21 |
|  | Others | 1 | (2.0%) | 1 | 0 |
|  | Dead at the time finding | 7 | (14.3%) | 5 | 2 |

dFAT; direct Fluorescent Antibody Test
