# Supplementary material for: Evaluation of a real-time mobile PCR device (PCR 1100) for the detection of the rabies gene in field samples
Source: Trop Med Health. 2023 Mar 17;51:17. doi: 10.1186/s41182-023-00501-3 (PMC10020757; doi:10.1186/s41182-023-00501-3)
Supplement: Supplementary file 2 — Additional file 2: Diagnostic results of experimentally infected mouse samples for different tissue. [file 41182_2023_501_MOESM2_ESM.docx]

Additional file 2: Diagnostic results of the experimentally infected mouse samples for different tissue.

|  | PCR1100 | | | | | | | |
| --- | --- | --- | --- | --- | --- | --- | --- | --- |
|  | Brain | |  | Muzzle skin | |  | Salivary gland | |
|  | ＋ | − |  | ＋ | − |  | ＋ | − |
| Rabid mouse | 6 | 0 |  | 6 | 0 |  | 3 | 3 |
| Mock | 0 | 4 |  | 0 | 4 |  | 0 | 4 |

+: detected, −: not detected
